# Personalized prognostic model for colorectal cancer in the era of precision medicine: a dynamic approach based on real-world data

**DOI:** 10.1007/s10147-025-02766-6

**Published:** 2025-05-01

**Authors:** Keisuke Okura, Keita Fukuyama, Satoru Seo, Hiroto Nishino, Tomoaki Yoh, Norihiro Shimoike, Takahiro Nishio, Yukinori Koyama, Satoshi Ogiso, Takamichi Ishii, Koya Hida, Shigemi Matsumoto, Manabu Muto, Satoshi Morita, Kazutaka Obama, Etsuro Hatano

**Affiliations:** 1 Department of Surgery, Graduate School of Medicine, Kyoto University, Kyoto, Japan; 2 Division of Medical Information Technology and Administration Planning, Kyoto University Hospital, 54 Kawahara-cho, Shogoin, Sakyo-ku, Kyoto, 606-8507 Japan; 3 Department of Surgery, Kochi Medical School, Kohasu, Okocho, Nankoku, Kochi, 783-8505 Japan; 4 Department of Real World Data Research and Development, Graduate School of Medicine, Kyoto University, Kyoto, Japan; 5 Department of Medical Oncology, Graduate School of Medicine, Kyoto University, Kyoto, Japan; 6 Department of Biomedical Statistics and Bioinformatics, Graduate School of Medicine, Kyoto University, Kyoto, Japan

**Keywords:** Colorectal cancer, Joint model, Dynamic prediction

## Abstract

**Background:**

Predicting individual prognosis is required for patients with colorectal cancer in the era of precision medicine. However, this may be challenging for the conventional survival analysis such as the Cox proportional hazards model. This study aims to develop a personalized prognostic prediction that incorporates longitudinal data to improve predictions for colorectal cancer patients.

**Methods:**

Patients with advanced or recurrent colorectal cancer, who received treatment at Kyoto University Hospital between April 2015 and December 2021, were retrospectively analyzed. The Joint model is one of the dynamic prediction models. Using longitudinal clinical data, a carcinoembryonic antigen (CEA) prediction equation was developed for each patient. Additionally, a personalized prognostic prediction model was created using the Joint model. The prediction accuracy of the Joint model was compared with one of the Cox proportional hazards model.

**Results:**

Among the 1010 patients, 614 patients were enrolled. The median frequency of tumor marker measurement (per patient) was 20 times (range: 3–117 times). CEA values could be predicted accurately and the Pearson’s correlation coefficient between measured CEA and predicted CEA was 0.931. In the Joint model, the significant prognostic factors were baseline age (HR, 1.039; 95% CI, 1.025–1.054), poor-differentiated tumor (HR, 2.600; 95% CI 1.446–4.675) and log_2_ (predicted CEA) (HR, 1.551; 95% CI 1.488–1.617). The areas under the curve at 2, 3, 4, and 5 were significantly higher for the Joint model than for the Cox proportional hazards model, respectively.

**Conclusion:**

The Joint model may accurately predict personalized prognosis that reflects changes in longitudinal tumor marker values.

**Supplementary Information:**

The online version contains supplementary material available at 10.1007/s10147-025-02766-6.

## Introduction

Colorectal cancer (CRC) is one of the deadliest cancers, ranking third in terms of incidence and second in terms of mortality [1]. The number of CRC patients is predicted to increase to 3.2 million new cases and 1.6 million deaths by 2040 [2].

Multidisciplinary treatment is the standard strategy, especially among patients with advanced or recurrent CRC [3]. Patients with clinical stage (cStage) IV CRC, even with unresectable liver metastasis, reportedly have a good prognosis if chemotherapy can lead to conversion surgery [4, 5]. However, there are various treatment strategies for CRC, and the decision-making process has become increasingly complex, especially in the era of precision medicine.

In the past, the primary method for prognostic analysis was the Cox proportional hazards model. However, as the prognosis for CRC has prolonged, accurately predicting prognosis using fixed-point clinical data has become increasingly challenging.

One possible solution is dynamic prediction. Incorporating new information into a prediction model allows for dynamic predictions, enabling the updated prognosis of patients [6]. However, there are few reports that examine the relationship between longitudinal tumor markers and prognoses. In the present study, we aimed to establish a personalized prognostic prediction model using longitudinal data.

## Patients and methods

### Ethics statement

This study was approved by Kyoto University Graduate School and Faculty of Medicine, Ethics Committee as R3856, and an opt-out consent method was used.

### Study population and design

Data obtained from patients with advanced or recurrent CRC, who received multimodal therapy through our multidisciplinary colorectal cancer unit team between January 2015 and December 2021, were retrospectively analyzed. The date of registration in the hospital-based cancer registry was defined as the start date, which was the date of referral to our hospital if the patients had already been diagnosed with colorectal cancer, or the date of diagnosis of colorectal cancer in our hospital. The exclusion criteria were as follows: (1) patients not registered in a hospital-based cancer registry; (2) patients with metachronous cancers before the start date; (3) patients with a histologic type different from adenocarcinoma; (4) patients with unfixed cStage; (5) patients with tumor marker measurements of less than three; and (6) patients undergoing hemodialysis at the start date.

**Fig. 1:**
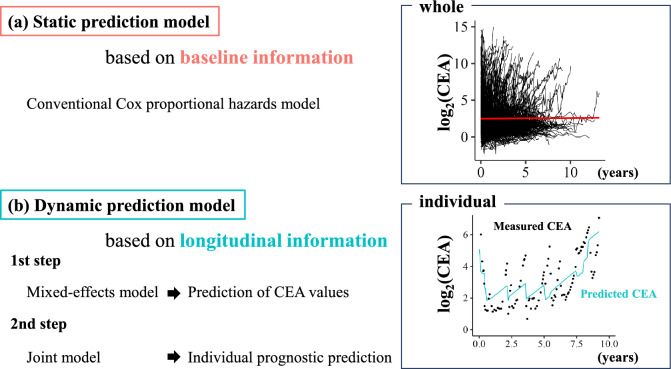
The study procedure. In this study, the comparison between static and dynamic prediction model was performed. In the dynamic prediction model, we first predicted the CEA values, using the mixed-effects model. Moreover, we analyzed individual prognosis, using the Joint model. In the upper figure, the black lines indicate the transition of measured CEA values for each patient, and the red line indicates a regression line. In the lower figure, the black points indicate the plot of measured CEA values for a specific patient, and the blue line indicates the transition of predicted CEA values by the mixed-effects model

We performed a prognostic analysis using both static and dynamic prediction models and compared the efficiency of each model (Fig. 1). The static prediction model was based on baseline information and the data were analyzed collectively, whereas the dynamic prediction model was based on longitudinal information. As described later, the Joint model has two steps [7] and allows an individual prognostic analysis.

### Data extraction

To comprehensively and efficiently collect patient information, the clinical data were extracted from the Data Warehouse (DWH; a secondary use database), receipt information (medical fee statements), and hospital-based cancer registry.

### Joint model

There are two basic components of the Joint model: the longitudinal component and the time-to-event component [8]. The mixed-effects model is employed to analyze the longitudinal component, and then the results are integrated into the Cox proportional hazards model for assessing survival prognosis. In the present study, the Joint model can incorporate longitudinal tumor markers and treatment history into the conventional Cox proportional hazards model. As the treatment history, the dosage of chemotherapy, the number of surgeries, and the number of radiation treatments were included. The carcinoembryonic antigen (CEA) values were expressed as a function of time *t* from the start date and the treatment history, based on the mixed-effects model. For predicting individual prognosis, the Joint model was employed to integrate both the predicted CEA values (CEA_predicted_) and the baseline information within the Cox proportional hazards model. The mathematical description and the clinical explanation of each model applied in the present study are presented in the supplementary document.

We extracted clinical information about baseline age, sex, cStage, tumor location, differentiation, and measured CEA values (CEA_measured_) from the electronic medical records (EMR). The list of variables is shown in Supplementary Table 1. Right-sided tumors were defined as the tumors originating from ileocecal, ascending, or transverse colon, whereas left-sided tumors were defined as the tumors originating from descending colon, sigmoid colon, or rectum [9]. In the conventional Cox proportional hazards model, the CEA values measured for the first time (CEA_1 st_) were used.

### Study outcomes

The main outcome was overall survival, which was defined as the period from the start date to the date of death or the last visit. In the conventional Cox proportional hazards model or the Joint model, a *p* value < 0.05 was considered statistically significant. As a predictive assessment of each model, the area under the curve (AUC) of the time-dependent receiver-operating characteristic (ROC) curve analysis was applied. It was a method that extended a typical ROC curve analysis to survival analysis [10, 11]. Specifically, the AUCs at 1, 2, 3, 4, and 5 years from the start date were compared. The differences in AUC for each year were examined using a paired permutation test under the null hypothesis that the outputs of the two models were equal. For CEA modeling, we also compared CEA_measured_ with CEA_predicted_ and provided typical case presentations.

### Analysis of environment

The analysis was conducted in a prebuilt environment using R version 4.2.3 (R Project for Statistical Computing)/Jupyter Lab version 3.6.6 (Project Jupyter) on Ubuntu 22.04.2 LTS on a closed network. The “JM” package was used for joint modeling [12]. The other packages used in this study are described in Supplementary Table 2. The codes are publicly available on GitHub (https://github.com/kokura-kuhp/CRC_dynamic_prediction).

## Results

### Characteristics of the patients in the study cohort

**Fig. 2:**
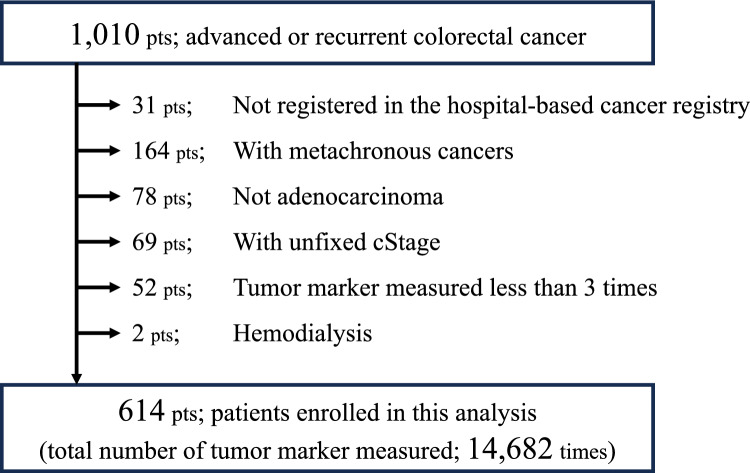
The study flowchart. *pts* patients

Altogether, 1010 patients with advanced or recurrent CRC who planned to undergo multidisciplinary treatment in our cancer board between April 2015 and December 2021 were included. After excluding ineligible patients, 614 patients were enrolled in the final analysis with a total of 14,682 tumor marker measurements collected (Fig. 2).

The patient characteristics are shown in Table 1. Among the enrolled patients, 356 were male participants and 258 were female participants, with a median age of 67 years (range: 22–91 years). The median number of tumor marker measurements in each patient was 20 times (range, 3–117 times). The number of patients who received each treatment is shown in Supplementary Table 3.

**Table 1:** The characteristics of patients with advanced or recurrent colorectal cancer

cStage	cStageI	cStageII	cStageIII	cStageIV	Total
	(*N* = 71)	(*N* = 97)	(*N* = 222)	(*N* = 224)	(*N* = 614)
Baseline age (years)					
Median [range]	67 [28–89]	69 [36–91]	68 [22–89]	64 [24–86]	67 [22–91]
Sex					
Male	34 (47.9%)	56 (57.7%)	135 (60.8%)	131 (58.5%)	356 (58.0%)
Female	37 (52.1%)	41 (42.3%)	87 (39.2%)	93 (41.5%)	258 (42.0%)
cT factor					
T1	35 (49.3%)	0 (0%)	5 (2.3%)	2 (0.9%)	42 (6.8%)
T2	36 (50.7%)	0 (0%)	14 (6.3%)	17 (7.6%)	67 (10.9%)
T3	0 (0%)	76 (78.4%)	97 (43.7%)	76 (33.9%)	249 (40.6%)
T4	0 (0%)	21 (21.6%)	106 (47.7%)	105 (46.9%)	232 (37.8%)
Unknown	0 (0%)	0 (0%)	0 (0%)	24 (10.7%)	24 (3.9%)
cN factor					
N0	71 (100%)	96 (99.0%)	0 (0%)	42 (18.8%)	209 (34.0%)
N1	0 (0%)	0 (0%)	160 (72.1%)	92 (41.1%)	252 (41.0%)
N2	0 (0%)	0 (0%)	62 (27.9%)	77 (34.4%)	139 (22.6%)
unknown	0 (0%)	1 (1.0%)	0 (0%)	13 (5.8%)	14 (2.3%)
cM factor					
M0	71 (100%)	97 (100%)	222 (100%)	0 (0%)	390 (63.5%)
M1	0 (0%)	0 (0%)	0 (0%)	224 (100%)	224 (36.5%)
Differentiation					
Well	26 (36.6%)	24 (24.7%)	46 (20.7%)	57 (25.4%)	153 (24.9%)
Moderate	44 (62.0%)	69 (71.1%)	163 (73.4%)	143 (63.8%)	419 (68.2%)
Poor	1 (1.4%)	4 (4.1%)	9 (4.1%)	14 (6.3%)	28 (4.6%)
unknown	0 (0%)	0 (0%)	4 (1.8%)	10 (4.5%)	14 (2.3%)
Location					
Ileocecal	1 (1.4%)	5 (5.2%)	5 (2.3%)	13 (5.8%)	24 (3.9%)
Ascending	5 (7.0%)	10 (10.3%)	30 (13.5%)	25 (11.2%)	70 (11.4%)
Transverse	5 (7.0%)	5 (5.2%)	16 (7.2%)	15 (6.7%)	41 (6.7%)
Descending	3 (4.2%)	6 (6.2%)	10 (4.5%)	5 (2.2%)	24 (3.9%)
Sigmoid	17 (23.9%)	21 (21.6%)	43 (19.4%)	70 (31.3%)	151 (24.6%)
Rectum	40 (56.3%)	50 (51.5%)	118 (53.2%)	95 (42.4%)	303 (49.3%)
Multiple	0 (0%)	0 (0%)	0 (0%)	1 (0.4%)	1 (0.2%)
CEA count (times)					
Median [range]	18 [3–109]	17 [4–65]	21 [3–105]	22 [3–117]	20 [3–117]

### Conventional Cox proportional hazards model

A multivariate analysis using the Cox proportional hazards model was performed using variables extracted from the EMR. The results are shown in Table 2. The significant prognostic factors were baseline age (*p* < 0.001, hazard ratio [HR] = 1.023, 95% CI 1.009–1.036), cStage (*p* = 0.011, HR = 1.677, 95% CI 1.125–2.500), poor-differentiated tumor (*p* = 0.018, HR = 2.018, 95% CI 1.129–3.609) and log_2_(CEA_1 st_) (*p* < 0.001, HR = 1.181, 95% CI 1.130–1.236).

**Table 2:** The estimated parameters for prognosis in the analysis using the Cox proportional hazards model

Variable		*p* value	HR	95% CI
Baseline age	Every 1-year increase	< 0.001	1.023	(1.009–1.036)
cStage	III or IV	0.011	1.677	(1.125–2.500)
	I, II, or unknown	Reference		
Differentiation	Poor	0.018	2.018	(1.129–3.609)
	Well, moderate, or unknown	Reference		
Sex	Male	0.524	1.102	(0.818–1.484)
	Female	Reference		
Location	Right-sided	0.548	1.120	(0.773–1.623)
	Left-sided	Reference		
log_2_(CEA_1 st_)	Every 1 increase	< 0.001	1.181	(1.130–1.236)

### Mixed-effects model

For the analysis in the Joint model, we first predicted the CEA values based on the treatment history. Using the mixed-effects model, we obtained each coefficient in the equation expressing CEA_predicted_ (Supplementary Document). The results are shown in Supplementary Table 4. It was possible to estimate the individual CEA_predicted_ at any time point based on the treatment history that changed over time.

Pearson’s correlation coefficient and mean squared error (MSE) were calculated to examine the predictions’ accuracy. Pearson’s correlation coefficient and mean MSE between CEA_measured_ and CEA_predicted_ were 0.931 (Supplementary Fig. 1) and 0.664, respectively.

### Joint model

The Joint model was also applied for the prognostic analysis based on the longitudinal information. The results are shown in Table 3. Baseline age (*p* < 0.001, HR = 1.039, 95% CI 1.025–1.054), poor-differentiated tumor (*p* = 0.001, HR = 2.600, 95% CI 1.446–4.675) and log_2_(CEA_predicted_) (*p* < 0.001, HR = 1.551, 95% CI 1.488–1.617) were the significant prognostic factors, but cStage (*p* = 0.958, HR = 1.011, 95% CI 0.672–1.522) was not.

**Table 3:** The estimated parameters for prognosis in the analysis using the joint model

Variable		*p* value	HR	95% CI
Baseline age	Every 1-year increase	< 0.001	1.039	(1.025–1.054)
cStage	III or IV	0.958	1.011	(0.672–1.522)
	I, II, or unknown	Reference		
Differentiation	Poor	0.001	2.600	(1.446–4.675)
	Well, Moderate, or unknown	Reference		
Sex	Male	0.691	0.941	(0.697–1.271)
	Female	Reference		
Location	Right-sided	0.418	1.168	(0.802–1.703)
	Left-sided	Reference		
log_2_(CEA_predicted_)	Every 1 increase	< 0.001	1.551	(1.488–1.617)

### Time-dependent ROC curve analysis

**Fig. 3:**
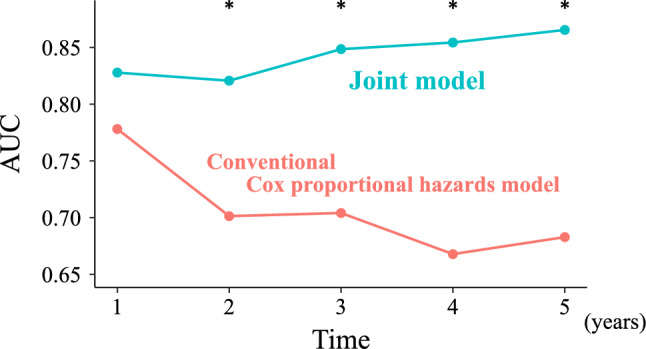
The AUCs calculated by time-dependent ROC in each model. The transition of AUCs calculated by time-dependent ROC in each model; The areas under the curve at 1, 2, 3, 4, and 5 years after the start date were 0.778, 0.701, 0.704, 0.668, and 0.683, respectively, for the conventional Cox proportional hazards model, and 0.828, 0.821, 0.849, 0.854, and 0.865, respectively, for the Joint model. The asterisk shows the significant difference between the two groups. *AUC* area under the curve, *ROC* receiver-operating characteristics

Dynamic and cumulative AUCs were calculated for each model through a time-dependent ROC curve analysis. The AUCs at 1, 2, 3, 4, and 5 years after the start date were 0.778, 0.701, 0.704, 0.668, and 0.683, respectively, for the conventional Cox proportional hazards model, and 0.828, 0.821, 0.849, 0.854, and 0.865, respectively, for the Joint model (Supplementary Fig. 2). The *p* values calculated by the paired permutation test at 1, 2, 3, 4, and 5 years were 0.477, 0.011, 0.002, < 0.001, and < 0.001, respectively. The AUCs of the Joint model significantly exceeded those of the Cox proportional hazards model at 2, 3, 4, and 5 years after the start date (Fig. 3).

### ***Clinical case presentation 1; the clinical course was illustrated in ***Fig. 4a

**Fig. 4:**
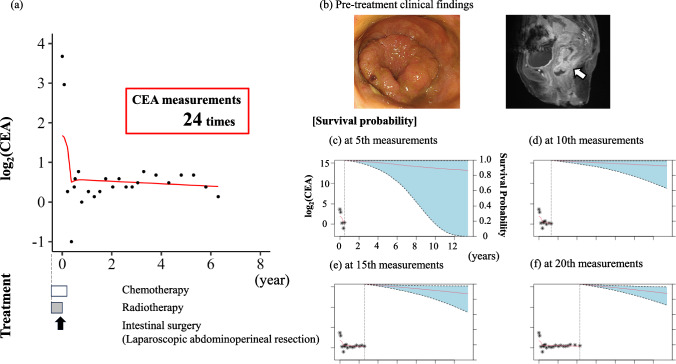
Clinical case presentation 1.** a** The longitudinal transition in log_2_(CEA). Black point indicates the measured log_2_(CEA). Red line indicates the predicted log_2_(CEA). White box indicates the chemotherapy duration. White arrows indicate the timing of liver surgery. Black arrow indicates the timing of intestinal surgery.** b** Pretreatment clinical findings. Endoscopic findings showed an obstructing colon cancer. Imaging findings showed an apple core sign at the rectum (white arrow).** c**–**f** Red line indicates the mean survival probability at each time point. Light blue area indicates the 95% CI for the survival curves. *CEA* carcinoembryonic antigen, *CI* confidence interval

A woman in her sixties was diagnosed with locally advanced rectal cancer and referred to our hospital after undergoing colostomy. The pretreatment findings are shown in Fig. 4b. The patient received preoperative chemoradiotherapy and underwent laparoscopic abdominoperineal resection 3 months after the initial visit. Postoperative adjuvant chemotherapy was administered, and the patient was alive for 7 years postoperatively without recurrence. The tumor markers were measured 24 times. These graphs show the survival probability at 5 th, 10 th, 15 th, and 20 th tumor marker measurements (Fig. 4c–f), indicating that the survival probability increased gradually as the tumor markers remained low. The transition of individual survival probability is shown as a movie in Supplementary Movie 1.

### ***Clinical case presentation 2; the clinical course was illustrated in ***Fig. 5a

**Fig. 5:**
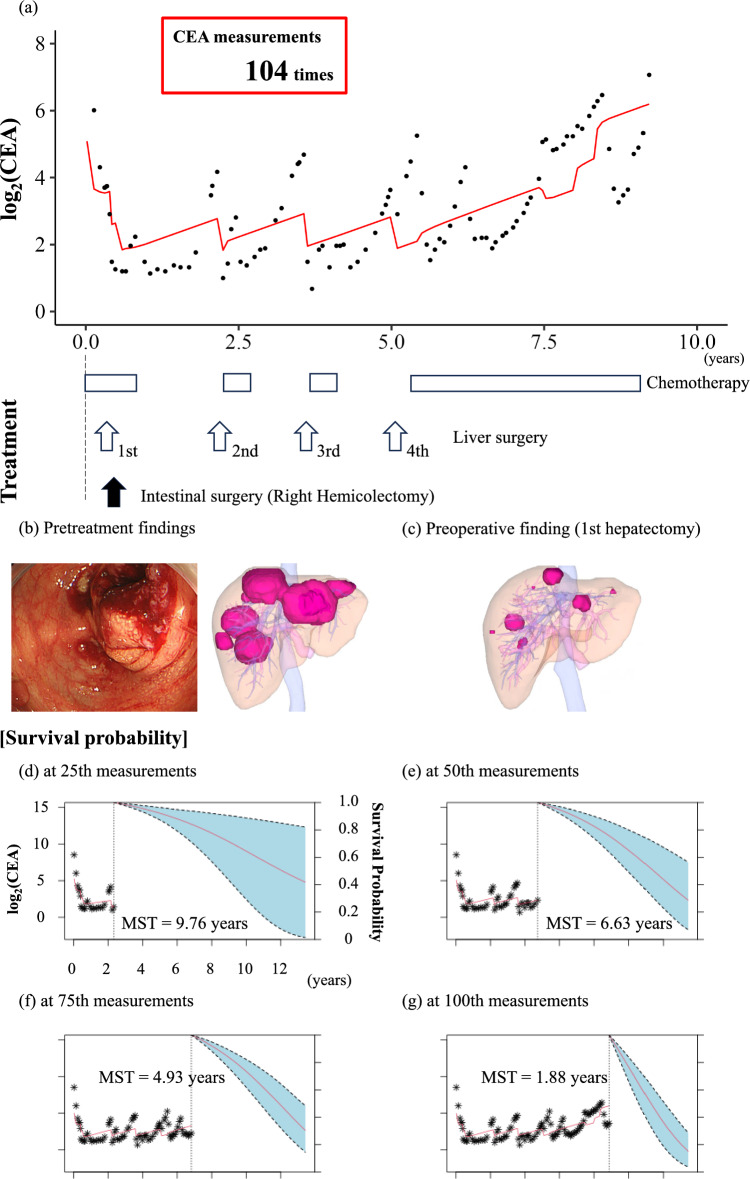
Clinical case presentation 2.** a** The longitudinal transition in log_2_(CEA). Black point indicates the measured log_2_(CEA). Red line indicates the predicted log_2_(CEA). White box indicates the chemotherapy duration. White arrows indicate the timing of liver surgery. Black arrow indicates the timing of intestinal surgery.** b** Pretreatment clinical findings. Endoscopic findings showed an obstructing colon cancer. Imaging findings, reconstructed by SYNAPSE VINCENT (Fujifilm Medical), showed multiple liver metastases. **c** Preoperative findings (first hepatectomy) showed that multiple liver metastases had shrunk remarkably after chemotherapy.** d**–**g** Red line indicates the mean survival probability at each time point. Light blue area indicates the 95% CI for the survival curves. *CEA* carcinoembryonic antigen, *CI* confidence interval, *MST* median survival time

A woman in her sixties was diagnosed with cT4 N2M1 cStage IV colon cancer, and her multiple liver metastases were judged to be unresectable (Fig. 5b). She then underwent systemic chemotherapy. The patient had a good treatment course (Fig. 5c) and underwent hepatectomy at 4.8 months after the diagnosis because her multiple liver metastases had shrunk remarkably. Six months after the diagnosis, her primary cancer was resected. The patient continued to have a recurrence of liver metastases, and hepatectomy was performed at 2.2, 3.6, and 5.1 years after the diagnosis. However, the tumor recurred and was judged to be unresectable. Subsequently, she underwent systemic chemotherapy and died 9.4 years after the diagnosis. The tumor markers were measured for a total of 104 times. These graphs show the survival probability at the 25 th, 50 th, 75 th, and 100 th tumor marker measurements (Fig. 5d–g). The disease progress was controlled favorably by the treatment but increased progressively over time, indicating a gradual decrease in survival probability. The transition of individual survival probability is shown as a movie in Supplementary Movie 2.

## Discussion

Prognostic analysis, which takes into account various clinical factors, is important in determining a patient’s treatment course. In the present study, longitudinal clinical data were systematically extracted from the EMR, and individual prognoses were analyzed using dynamic prediction. This study demonstrated that (1) the analytic system could be established by incorporating the longitudinal clinical data into the Joint model and (2) the longitudinal CEA value was a significant prognostic factor. We also proposed (3) a novel, individualized prognostic prediction model. Conversely, we demonstrated that cStage, which was dichotomized (cStage I and II vs. III and IV) based on prognosis, was a significant prognostic factor in the conventional Cox proportional hazards model, but not in the Joint model. The AUCs of the time-dependent ROC curves at 2, 3, 4, and 5 years were significantly higher for the Joint model than for the conventional Cox proportional hazards model. These results suggested the importance of continuously monitoring patients. Nowadays, the approaches of genomic analysis and biomarker research have shown advancements daily [13, 14], and we need to implement personalized strategies through the integration of the latest information. Long-term survival will be expected in advanced or recurrent CRC if the disease status is controlled [15]. The Joint model offers accurate personalized prognosis using longitudinal clinical data, and it could be one of the key components for prognosis prediction in the era of precision medicine. Some trials demonstrated that the association between OS and RFS has been declining for patients with CRC [16], and establishing the appropriateness of this surrogate endpoint for OS is urgently needed [17]. The results suggest that monitoring CEA values could be one of the solutions to this problem.

The conventional Cox proportional hazards model is still the mainstream of prognostic analysis. However, only the baseline clinical data can be included in this model, which is a significant discrepancy from daily clinical practice [18]. This is why we focused on the time course of tumor marker levels. First, we reported the impact of the uptrend of the preoperative CEA values [19], but the change at two time points could not accurately predict the patient’s prognosis. Next, we performed a dynamic prediction using various structured medical records. Longitudinal tumor marker measurements have been reported to be important for predicting the prognosis or recurrence of patients with CRC [20, 21]. The present study is the first attempt to apply a mathematical model with multimodal treatment history to prognosis prediction. The time-dependent ROC curve analysis showed a better prognostic performance for the Joint model than for the conventional Cox proportional hazards model, indicating the importance of considering the treatment strategy based on the individual’s clinical course.

In the present study, a mixed-effects model was applied to predict the CEA values. The Joint model could calculate survival probability based on the longitudinal CEA values. The coefficients of each treatment were expressed in Supplementary Table 4. The estimated coefficients do not allow for a discussion on the effectiveness of each treatment because the combination effects of various chemotherapeutic agents or multimodal treatments were not considered in this study. However, based on these results, CEA simulation with treatment history can be performed for new patients with CRC. To guide decision-making based on the clinical course, quantitatively representing the impact of each treatment and accurately clarifying the individual mortality are important [22].

The clinical data used in the present study included treatment history data and more than 10,000 blood test measurements, which were used as variables. These are beyond the scale of data sets that can be extracted manually from the EMR. The strong point of this study lies in the elimination of any manual data extraction. Currently, clinical data have been accumulated by integrating many hospitals on various platforms, and programmatic approaches for larger data sets are expected to increase soon due to the ambiguity introduced by manual data entry [23]. In future, we hope to integrate this system into EMR to facilitate it in clinical practice. Once implemented, this analysis could be performed automatically.

There are several limitations to this study. First, this analysis is based on the assumption that CEA values reflect the disease status in CRC. CEA values were selected because the sensitivity of serum CA19-9 may be low [24]. Moreover, this study was limited to patients with advanced or recurrent CRC to focus on CRC-specific death. The second limitation is the feasibility of treatment. Based on our results, the prognosis of patients with CRC cannot be completely predicted because it does not show whether the patient can tolerate each treatment. The third limitation is the poor variable setting. In this study, the clinical data were systematically extracted from the EMR, with respect to the accessibility of the data. Consequently, not all prognostic factors could be incorporated. Unstructured data, including image findings or PDF-formatted data, could not be extracted in this study. More detailed oncologic information will be required for further research. Fourth, the present investigation was a retrospective single-center study in a tertiary university hospital, which might introduce some biases. Therefore, it is not appropriate to apply the results to other facilities. However, the study methodology is applicable to other institutions with similar structured data and allows for the construction of validation data sets without manual data extraction. Based on this methodology, we expect that prospective, multicenter studies will be more meaningful by constructing the simulation system within the EMR.

## Conclusion

The Joint model analyzed the prognostic factors of patients with CRC based on longitudinal clinical information, and this model can establish a novel prognostic prediction tailored to individuals with CRC.

## Supplementary Information

Below is the link to the electronic supplementary material.

Supplementary file 1 (DOCX 292 KB)

Supplementary file 2 (PPTX 178 KB)

Supplementary file 3 (PPTX 116 KB)

Supplementary file 4 (MP4 1018 KB)

Supplementary file 5 (MP4 3928 KB)

Supplementary file 6 (DOCX 30 KB)

Supplementary file 7 (DOCX 30 KB)

Supplementary file 8 (DOCX 31 KB)

Supplementary file 9 (DOCX 30 KB)

## Data Availability

The datasets generated and analyzed during the current study are not publicly available due to ethical concerns but can be obtained from the corresponding author upon reasonable request.
