# Supplementary material for: Personalized prognostic model for colorectal cancer in the era of precision medicine: a dynamic approach based on real-world data
Source: Int J Clin Oncol. 2025 May 1;30(7):1376–85. doi: 10.1007/s10147-025-02766-6 (PMC12187870; doi:10.1007/s10147-025-02766-6)
Supplement: Supplementary file 1 — (DOCX 292 KB) [file 10147_2025_2766_MOESM1_ESM.docx]

**Supplementary Document**

**Mathematical description**

Case index:

$$i (1\leq i\leq614, i\in\boldsymbol{N})$$

Baseline variables in case *i* (such as baseline age, cStage, sex, CEA_1st_, etc.), where *j* indicates each baseline variable:

$$X_{i}=\left( x_{{age}_{i}}, x_{{sex}_{i}}, \cdots, x_{j_{i}}, \cdots, x_{{CEA}_{1st i}} \right)$$

Longitudinal variables in case *i* at time *t* (such as a period from the start date, and accumulated treatments), where *k* indicates each longitudinal variable:

$$Y_{i}\left( t \right) = \left( {y_{{time}_{i}}\left( t \right),y_{dose_{{chemo}_{i}}}\left( t \right), \cdots, y}_{{liver\_surgery}_{i}}\left( t \right), \cdots, y_{{radio}_{i}}, \cdots, y_{k_{i}}\left( t \right), \cdots\right)$$

The Cox proportional hazard model at baseline variables for case *i*:

$$hi\left( t|X_{i} \right) =h_{0}\left( t \right)\cdot exp\left( \beta_{0}+\beta_{sex}\cdot x_{{sex}_{i}}+\cdots+\beta_{{CEA}_{1st}}\cdot x_{{CEA}_{1st i}} \right)$$

$$= h_{0}\left( t \right)\cdot\exp\left( \sum_{j} \beta_{j}\cdot x_{j_{i}} \right)$$

The mixed-effects model about longitudinal marker measurements in case *i* at time *t*, where *ε* is a measurement error. Random effects were estimated only for the slope to time, b_1_*_i_*, and the intercept, b_0_*_i_*. CEA values converted to log_2_ were applied as the marker value:

$${CEA}_{{measured}_{i}}\left( t \right)= {CEA}_{{predicted}_{i}}\left( t \right)+ \varepsilon_{i}\left( t \right)$$

$${CEA}_{{hidden}_{i}}\left( t \right)= b_{0_{i}}+b_{1_{i}}\cdot y_{{time}_{i}}\left( t \right)+\left\{ \sum_{j} b_{j_{i}}\cdot y_{j_{i}}(t) \right\}$$

The Joint model predicts patient survival based on baseline variables (excluding CEA_1st_) and longitudinal variables:

$$h_{i}\left( t|X_{i}, Y_{i}\left( t \right) \right) =h_{0}\left( t \right)\cdot\exp\left\{ \sum_{j} \beta_{j}\cdot x_{j_{i}}+\alpha\cdot{CEA}_{{hidden}_{i}}\left( t \right) \right\} \left( j\neq{CEA}_{1st} \right)$$

**Clinical explanation**

1. Clinical explanation
   1. Cox proportional hazard model at baseline for each patient (case *i*):


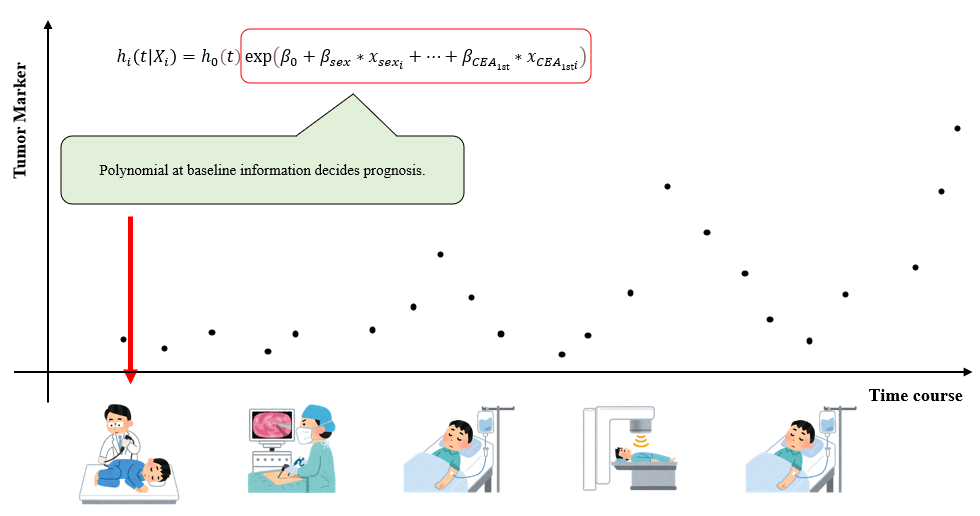


This model predicts patients’ prognosis as polynomial from baseline information. This method can’t integrate the time course with treatment and tumor makers reflecting cancer progression after baseline information.

- 1. Mixed effects sub model about longitudinal maker measure in case *i* at time *t*, where *ε* is measurement error:


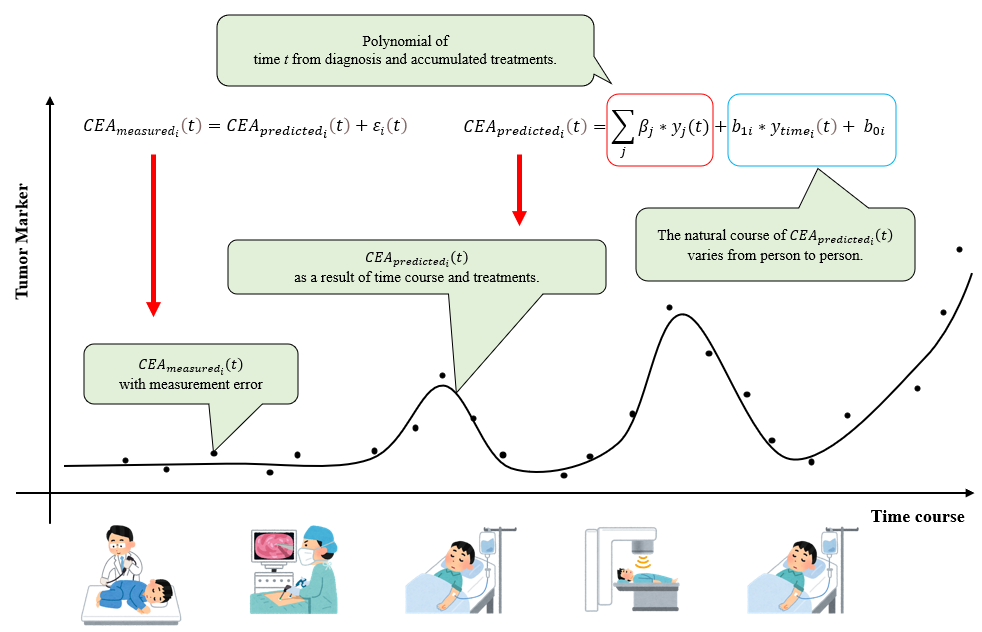


We estimated random effects only on slope to time and intercept, b_1_*_i_* and b_0_*_i_* respectively. This model predicts CEA values as a result of time course and accumulated treatments with the assumption that there is measurement error and natural course of tumor maker varies from person to person.

- 1. Joint model predicts patient survival from baseline variable and longitudinal variable.


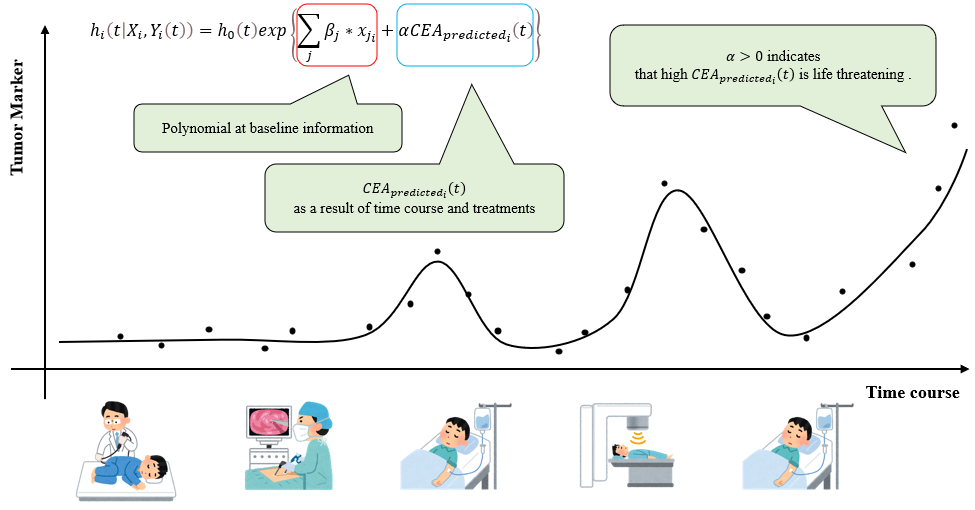


This model predicts patient survival from baseline information and longitudinal variables via predicted tumor marker values.
