# Supplementary figures and images for: Personalized prognostic model for colorectal cancer in the era of precision medicine: a dynamic approach based on real-world data
Source: Int J Clin Oncol. 2025 May 1;30(7):1376–85. doi: 10.1007/s10147-025-02766-6 (PMC12187870; doi:10.1007/s10147-025-02766-6)

## Slide 1
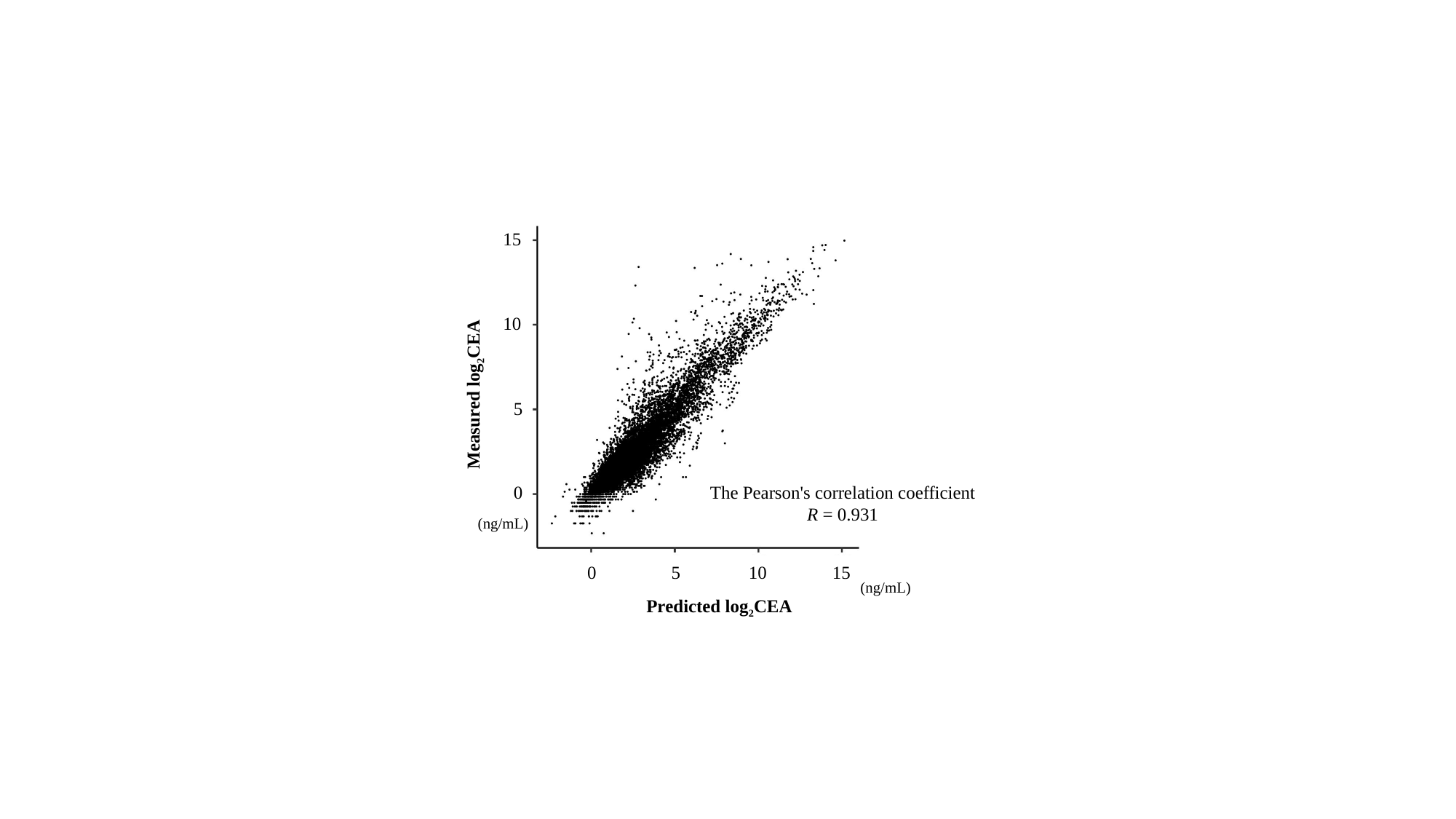

15
10
Measured log2CEA
5
0
The Pearson's correlation coefficient
 R = 0.931
(ng/mL)
0
5
10
15
(ng/mL)
Predicted log2CEA

Supplement: Supplementary file 2 — (PPTX 178 KB) [file 10147_2025_2766_MOESM2_ESM.pptx]

## Slide 1
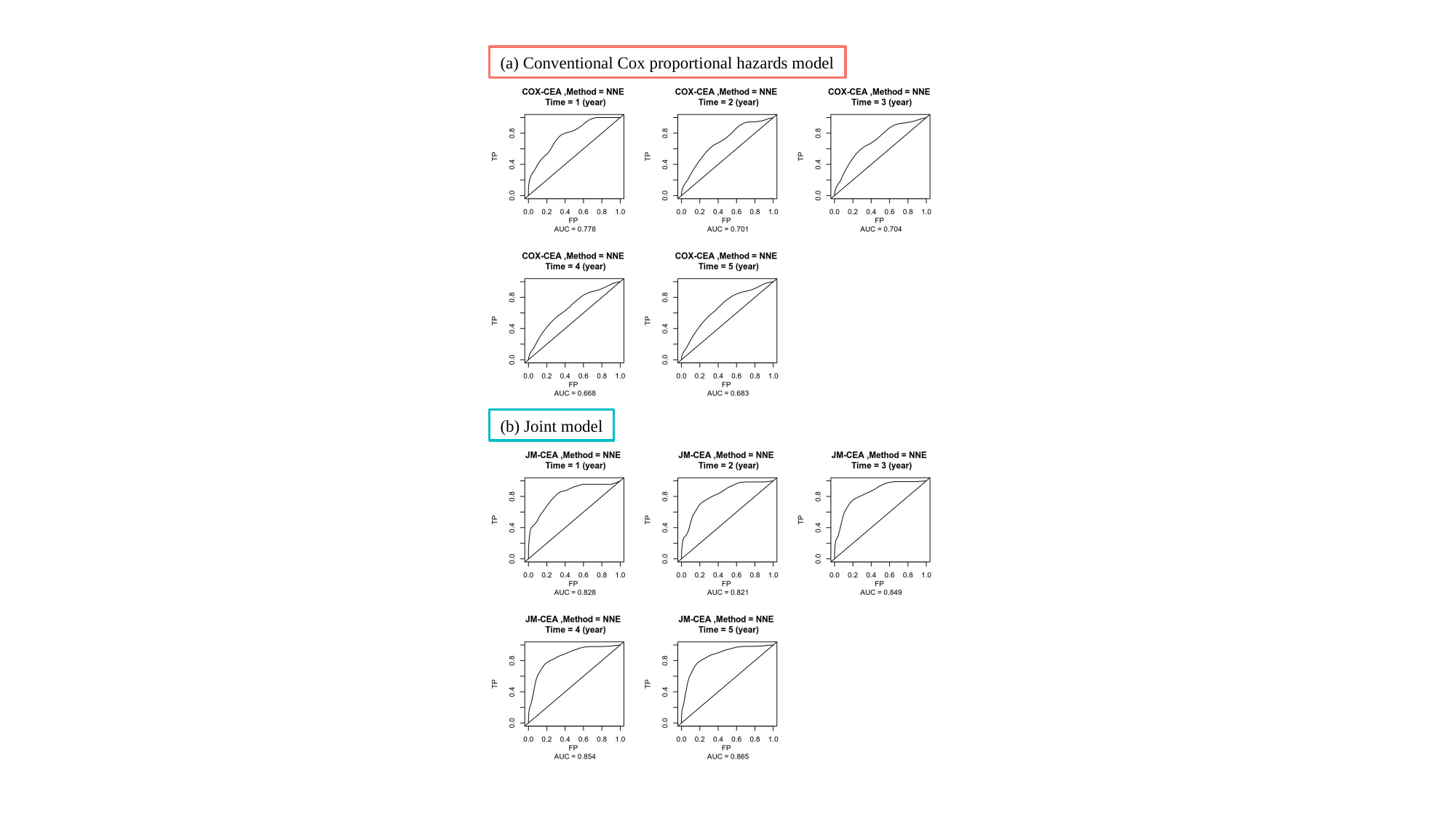

(a) Conventional Cox proportional hazards model
(b) Joint model

Supplement: Supplementary file 3 — (PPTX 116 KB) [file 10147_2025_2766_MOESM3_ESM.pptx]
