# Supplementary material for: Personalized prognostic model for colorectal cancer in the era of precision medicine: a dynamic approach based on real-world data
Source: Int J Clin Oncol. 2025 May 1;30(7):1376–85. doi: 10.1007/s10147-025-02766-6 (PMC12187870; doi:10.1007/s10147-025-02766-6)
Supplement: Supplementary file 6 — (DOCX 30 KB) [file 10147_2025_2766_MOESM6_ESM.docx]

| **Supplementary Table 1**  **The explanation of the variables in this study** | |
| --- | --- |
| **Variable** | **Explanation** |
| Baseline age | age at the time of initial visit or diagnosis. |
| cStage | 1 if III or IV, 0 if not. clinical Stage is based on The TNM Classification of Malignant Tumours. |
| Differentiation | 1 if poor-differentiated, 0 if not. |
| Sex | 1 if male, 0 if female. |
| Location | 1 if right-sided tumor, 0 if left-sided tumor. |
