# Supplementary material for: Personalized prognostic model for colorectal cancer in the era of precision medicine: a dynamic approach based on real-world data
Source: Int J Clin Oncol. 2025 May 1;30(7):1376–85. doi: 10.1007/s10147-025-02766-6 (PMC12187870; doi:10.1007/s10147-025-02766-6)
Supplement: Supplementary file 7 — (DOCX 30 KB) [file 10147_2025_2766_MOESM7_ESM.docx]

| **Supplementary Table 2**  **The list of R packages used in this study** | |
| --- | --- |
| **Package** | **Purpose** |
| data.table | data handling |
| JM | analysis for joint model |
| ggpubr | visualization |
| officer | visualization |
| openxlsx | data analysis |
| nlme | analysis for mixed-effects model |
| survival | analysis for survival |
| survivalROC | analysis for model performance evaluation |
| tidyverse | data handling |
