# Supplementary material for: Personalized prognostic model for colorectal cancer in the era of precision medicine: a dynamic approach based on real-world data
Source: Int J Clin Oncol. 2025 May 1;30(7):1376–85. doi: 10.1007/s10147-025-02766-6 (PMC12187870; doi:10.1007/s10147-025-02766-6)
Supplement: Supplementary file 8 — (DOCX 31 KB) [file 10147_2025_2766_MOESM8_ESM.docx]

| **Supplementary Table 3**  **The number of patients received each treatment** | | |
| --- | --- | --- |
| **Variable** | **Treatment** | **Number (%)** |
| inj_AFL | Aflibercept | 12 (2.0) |
| inj_Bev | Bevacizumab | 177 (28.8) |
| inj_Cmab | Cetuximab | 76 (12.4) |
| inj_FU_LV | 5-Fluorouracil, Leucovorin | 202 (32.9) |
| inj_HER | Trastuzumab | 0 (0) |
| inj_IRI | Irinotecan | 205 (33.4) |
| inj_Nivo | Nivolumab | 1 (0.2) |
| inj_OHP | Oxaliplatin | 316 (51.5) |
| inj_Pembro | Pembrolizumab | 1 (0.2) |
| inj_Pmab | Panitumumab | 95 (15.5) |
| inj_Rmab | Ramucirumab | 13 (2.1) |
| prs_BINI | Binimetinib | 1 (0.2) |
| prs_Cape | Capecitabine | 246 (40.1) |
| prs_REG | Regorafenib | 34 (5.5) |
| prs_TAS102 | Trifluridine Tipiracil Hydrochloride | 71 (11.6) |
| prs_TS1 | Tegafur Gimeracil Oteracil Potassium | 121 (19.7) |
| prs_UFT | Tegafur Uracil | 38 (6.2) |
| si_colrec_sx | Intestinal surgery | 490 (79.8) |
| si_endo_tx | Endoscopic therapy | 107 (17.4) |
| si_liv_sx | Liver surgery | 136 (22.1) |
| si_lng_sx | Lung surgery | 76 (12.4) |
| si_rtx | Radiotherapy | 141 (23.0) |
