# Supplementary material for: Personalized prognostic model for colorectal cancer in the era of precision medicine: a dynamic approach based on real-world data
Source: Int J Clin Oncol. 2025 May 1;30(7):1376–85. doi: 10.1007/s10147-025-02766-6 (PMC12187870; doi:10.1007/s10147-025-02766-6)
Supplement: Supplementary file 9 — (DOCX 30 KB) [file 10147_2025_2766_MOESM9_ESM.docx]

| **Supplementary Table 4**  **The estimated coefficients in analysis with mixed-effects model** | |
| --- | --- |
| **Variable** | **Coefficient** |
| inj_AFL | 0.172 |
| inj_Bev | -0.034 |
| inj_Cmab | -0.061 |
| inj_FU_LV | -0.033 |
| inj_IRI | 0.013 |
| inj_Nivo | 0.394 |
| inj_OHP | -0.063 |
| inj_Pembro | -0.081 |
| inj_Pmab | 0.012 |
| inj_Rmab | 0.039 |
| prs_BINI | 0.163 |
| prs_Cape | 0.016 |
| prs_REG | 0.059 |
| prs_TAS102 | 0.104 |
| prs_TS1 | 0.018 |
| prs_UFT | -0.005 |
| si_colrec_sx | -0.869 |
| si_endo_tx | 0.042 |
| si_liv_sx | -1.004 |
| si_lng_sx | -0.268 |
| si_rtx | -0.024 |
| time | 0.628 |
